# SARS-CoV-2 Antigens Expressed in Plants Detect Antibody Responses in COVID-19 Patients

**DOI:** 10.3389/fpls.2021.589940

**Published:** 2021-03-31

**Authors:** Mohau S. Makatsa, Marius B. Tincho, Jerome M. Wendoh, Sherazaan D. Ismail, Rofhiwa Nesamari, Francisco Pera, Scott de Beer, Anura David, Sarika Jugwanth, Maemu P. Gededzha, Nakampe Mampeule, Ian Sanne, Wendy Stevens, Lesley Scott, Jonathan Blackburn, Elizabeth S. Mayne, Roanne S. Keeton, Wendy A. Burgers

**Affiliations:** ^1^Institute of Infectious Disease and Molecular Medicine, University of Cape Town, Cape Town, South Africa; ^2^Division of Medical Virology, Department of Pathology, University of Cape Town, Cape Town, South Africa; ^3^Cape Bio Pharms, Cape Town, South Africa; ^4^Department of Molecular Medicine and Haematology, University of Witwatersrand, Johannesburg, South Africa; ^5^Department of Immunology, Faculty of Health Sciences, University of Witwatersrand and National Health Laboratory Service, Johannesburg, South Africa; ^6^Clinical HIV Research Unit, Department of Internal Medicine, University of Witwatersrand, Johannesburg, South Africa; ^7^Division of Chemical and Systems Biology, Department of Integrative Biomedical Sciences, University of Cape Town, Cape Town, South Africa; ^8^Wellcome Centre for Infectious Diseases Research in Africa, University of Cape Town, Cape Town, South Africa

**Keywords:** SARS-CoV-2, COVID-19, serology, ELISA, plant expression

## Abstract

**Background**: The severe acute respiratory syndrome coronavirus 2 (SARS-CoV-2) pandemic has swept the world and poses a significant global threat to lives and livelihoods, with 115 million confirmed cases and at least 2.5 million deaths from Coronavirus disease 2019 (COVID-19) in the first year of the pandemic. Developing tools to measure seroprevalence and understand protective immunity to SARS-CoV-2 is a priority. We aimed to develop a serological assay using plant-derived recombinant viral proteins, which represent important tools in less-resourced settings.

**Methods**: We established an indirect ELISA using the S1 and receptor-binding domain (RBD) portions of the spike protein from SARS-CoV-2, expressed in *Nicotiana benthamiana*. We measured antibody responses in sera from South African patients (*n* = 77) who had tested positive by PCR for SARS-CoV-2. Samples were taken a median of 6 weeks after the diagnosis, and the majority of participants had mild and moderate COVID-19 disease. In addition, we tested the reactivity of pre-pandemic plasma (*n* = 58) and compared the performance of our in-house ELISA with a commercial assay. We also determined whether our assay could detect SARS-CoV-2-specific IgG and IgA in saliva.

**Results:** We demonstrate that SARS-CoV-2-specific immunoglobulins are readily detectable using recombinant plant-derived viral proteins, in patients who tested positive for SARS-CoV-2 by PCR. Reactivity to S1 and RBD was detected in 51 (66%) and 48 (62%) of participants, respectively. Notably, we detected 100% of samples identified as having S1-specific antibodies by a validated, high sensitivity commercial ELISA, and optical density (OD) values were strongly and significantly correlated between the two assays. For the pre-pandemic plasma, 1/58 (1.7%) of samples were positive, indicating a high specificity for SARS-CoV-2 in our ELISA. SARS-CoV-2-specific IgG correlated significantly with IgA and IgM responses. Endpoint titers of S1- and RBD-specific immunoglobulins ranged from 1:50 to 1:3,200. S1-specific IgG and IgA were found in saliva samples from convalescent volunteers.

**Conclusion**: We demonstrate that recombinant SARS-CoV-2 proteins produced in plants enable robust detection of SARS-CoV-2 humoral responses. This assay can be used for seroepidemiological studies and to measure the strength and durability of antibody responses to SARS-CoV-2 in infected patients in our setting.

## Introduction

The current global pandemic, caused by the novel severe acute respiratory syndrome coronavirus 2 (SARS-CoV-2), has resulted in over 115 million cases and at least 2.5 million deaths, as of 02 March 2021. SARS-CoV-2 was first detected in December 2019 in Wuhan, a city in the Hubei province of China, and is thought to originate from zoonotic transmission of a bat coronavirus (Tan et al., 2020; Zhu et al., 2020). Coronavirus disease 2019 (COVID-19), the resultant disease, is commonly associated with fever, cough, and fatigue, and in severe cases, pneumonia and respiratory failure (Chan et al., 2020).

SARS-CoV-2 is a 30 kB positive-stranded RNA virus that is a member of the *Betacoronavirus* genus and the subgenus *Sarbecovirus* (Letko et al., 2020). The genus harbors human pathogens that cause respiratory infections, namely the highly virulent SARS-CoV and Middle East respiratory syndrome coronavirus (MERS-CoV), as well as the circulating “common cold” human coronavirus (hCoV)-OC43 and hCoV-HKU1 (Su et al., 2016). Betacoronaviruses express four essential structural proteins, namely the spike (S) glycoprotein, membrane (M) protein, envelope (E) protein, and nucleocapsid (N) protein, as well as multiple accessory and non-structural proteins (Neuman et al., 2011; Lu et al., 2020). The S glycoprotein is a homotrimer that protrudes from the surface of the viral particles (Tortorici and Veesler, 2019), and interacts with the human cell receptor angiotensin converting enzyme 2 (ACE2) through the receptor-binding domain (RBD), gaining viral entry into the host cell (Li, 2016; Letko et al., 2020; Walls et al., 2020). S is cleaved by host cell proteases into two subunits: the S1 subunit which harbors the RBD and enables binding to host cell receptors, and the S2 subunit that is important for fusion with the host cell membrane (Walls et al., 2020; Wrapp et al., 2020).

The S1 subunit is highly immunogenic, and its RBD portion is the main target of neutralizing antibodies, thus becoming the focus of serological studies (Amanat et al., 2020; Huang et al., 2020; Liu et al., 2020; Okba et al., 2020). Recently, potent neutralizing antibodies isolated from the convalescent sera of SARS-CoV-2 patients were demonstrated to be protective against disease from high-dose SARS-CoV-2 challenge in a small animal model (Rogers et al., 2020), suggesting the potential for therapeutic interventions as well as inferring that recovered SARS-CoV-2 patients may be afforded protection from re-infection by neutralizing antibody responses. Amanat et al. (2020) showed a strong correlation between the neutralizing antibody response and ELISA endpoint titers against S, suggesting the use of serological assays in estimating the percentage of infected people who have neutralizing antibodies that protect them from re-infection or disease.

Serological assays that can detect antibody responses to SARS-CoV-2 are critical for answering pressing questions regarding immunity to the virus. It is not known what proportion of infected individuals elicit antibodies to SARS-CoV-2, if antibodies serve as correlates of protection, and if so, what the threshold of binding or neutralizing titers are that will provide immunity, and the duration of these responses. Serological assays such as ELISA can assist in answering these questions. These assays need to be both sensitive as well as demonstrate high specificity for SARS-CoV-2, and not give false positives due to cross-reactivity with widely circulating hCoVs NL63, 229E, OC43, and HKU1. While the N protein is more conserved among coronaviruses, the S protein sequence has lower sequence conservation. The S1 portion is 21–25% identical at the amino acid level to circulating hCoVs (Okba et al., 2020). Thus, serological assays using the full-length S protein, S1 subunit, or RBD portion as antigens have shown good specificity with little cross-reactivity to NL63 and 229E (Amanat et al., 2020; Rosales-Mendosa et al., 2020) compared to the use of N protein (Rosales-Mendosa et al., 2020).

Purified recombinant proteins are essential for the establishment of serological assays. Numerous protein expression systems exist, each with their own advantages and limitations. These include bacterial, mammalian, yeast, insect, and plant-based systems (Yin et al., 2007; Shanmugaraj et al., 2020). Plant-based systems have several advantages over more widely used conventional protein expression systems. Most notably, they are rapid, cost-effective, and support post-translational modifications similar to mammalian cell systems, making them attractive protein expression systems particularly in low-income settings (Maliga and Graham, 2004; Shanmugaraj et al., 2020). Historically, their major disadvantage was low yield (Shanmugaraj et al., 2020); however, advances in plant technology, including transient expression systems and viral vectors, have led to improvements in protein yield (Kapila et al., 1997; Yamamoto et al., 2018). Additionally, SARS-CoV S1 protein expressed in tomato and tobacco plants demonstrated good immunogenicity in mice (Pogrebnyak et al., 2005). Together, these studies highlight the potential of plant-based expression systems for the development of serological assay reagents as well as vaccines for the current SARS-CoV-2 pandemic.

In this study, we describe the development of an ELISA that enables detection of antibodies directed at the S1 subunit and the RBD portion of the SARS-CoV-2 S glycoprotein, generated through a plant-based expression system.

## Materials and Methods

### Cloning and Expression of Recombinant Proteins

The S1 portion and receptor binding domain (RBD) of the spike protein of SARS-CoV-2 Wuhan-Hu-1 isolate (GenBank: MN908947.3) were produced by Cape Bio Pharms, Cape Town, South Africa. Briefly, *Nicotiana benthamiana* codon-optimized DNA encoding S1 (aa 14–698) and an extended region containing the RBD (aa 281–698) were synthesized commercially (Genscript). Both genes were fused at their C-terminal region to the fragment crystallizable region (Fc) of rabbit IgG1 (Genbank: L29172.1) and subsequently cloned into Cape Bio Pharms’ proprietary vector, pCBP2. *Agrobacterium tumefaciens* strain GV3101 (pMP90RK) was used to carry agroinfiltration. Growth of recombinant *A. tumefaciens* and vacuum infiltration of *N. benthamiana* plants were performed as described previously (Maclean et al., 2007). Three days post-infiltration, leaves were homogenized in the presence of phosphate buffered saline (PBS) at a 2:1 ratio buffer:leaf material. Cell debris was removed by centrifugation at 10,000 *g* for 10 min at 4°C, and the clarified supernatant was used for expression analyses and purification by Protein A affinity chromatography.

For purification, the extract was filtered through a 0.22 μm cellulose nitrate filter (Sartorius) before loading onto a pre-equilibrated 5 ml column packed with POROS MabCapture A resin (Thermo Fisher). The column was then washed with 10 column volumes of wash buffer (PBS, pH 7.5) and bound proteins eluted using elution buffer (0.1 M glycine, pH 2.5). Eluted fractions were captured in 1/10th volume of neutralization buffer (1 M Tris, pH 8.5) and then pooled and applied to a 10 K molecular weight cutoff Amicon centrifuge tube (Millipore) for buffer exchange against PBS and sample concentration.

### SDS-PAGE and Western Blot

Expression and purity of recombinant S1 and RBD fusion proteins were evaluated by western blot and sodium dodecyl sulfate-polyacrylamide gel electrophoresis (SDS-PAGE). Purified samples were added to sample loading dye NuPAGE LDS sample buffer and reducing agent (both Invitrogen) and heated to 70°C for 10 min. Samples were loaded into pre-cast polyacrylamide gels (Bolt 4–12% Bis-Tris Plus; Invitrogen) and run at 200 V for 40 min. Visualization of protein samples on acrylamide gels was performed using Coomassie Brilliant Blue G250 stain (Merck). Gels were stained overnight with agitation, and destaining solution (30% methanol and 10% acetic acid) was added for 1 h at room temperature. After separation by SDS-PAGE, proteins were transferred to a nitrocellulose membrane using a dry transblotter (Invitrogen). The membrane was blocked for 30 min (PBS containing 5% fat free milk and 0.1% Tween 20) at room temperature, followed by incubation with mouse anti-rabbit horseradish peroxidase (1:5,000; Sigma) for 1 h at 37°C with agitation. The membrane was washed four times using wash buffer (PBS with 0.1% Tween 20) at room temperature for 15 min, and developed using TMB solution (1-step ultra TMB-blotting solution, Thermo Scientific) in the dark for 30 min.

### Volunteer Recruitment and Sample Collection

Samples were collected from SARS-CoV-2 infected volunteers (*n* = 77) recruited from Gauteng and the Western Cape provinces of South Africa from 10 April 2020 to 26 May 2020. Volunteers had previously undergone a reverse transcriptase PCR (RT-PCR) test for SARS-CoV-2 from an upper respiratory tract (nose/throat) swab collected into viral transport media. Swabs were processed through approved assays in accredited public and private clinical laboratories. Inclusion criteria were age >=18 years and a confirmed positive PCR for SARS-CoV-2 on the national database of the National Health Laboratory Services (NHLS). Of the 77 participants, 34 (44%) had a second positive PCR result recorded within a week after the first positive test. With respect to disease severity, five participants were asymptomatic, 23 had mild disease (characterized by mild upper respiratory tract symptoms), 38 had moderate disease (defined by gastrointestinal symptoms or lower respiratory tract symptoms), and two had severe disease (admission to hospital). Serum and saliva samples were collected between 8 and 70 days after the first positive PCR test. An additional 101 volunteers who had a negative RT-PCR test were included in the Euroimmun testing (described below). Ethical approval for these studies was obtained from the Human Research Ethics Committee (HREC) of the University of Witwatersrand (M200468) and the University of Cape Town (210/2020). All participants provided written, informed consent.

Pre-pandemic plasma (*n* = 58) was obtained from banked human samples that were collected from participants recruited from Cape Town, South Africa in 2011–2012, from a study protocol approved by the HREC of the University of Cape Town (158/2010). Storage consent was provided by all participants, and approval for use of the samples in this study was obtained from the HREC, UCT. Samples came from participants who were HIV-infected (*n* = 27) or HIV-uninfected (*n* = 31). All participants had tested positive for exposure to *Mycobacterium tuberculosis* based on a positive IFN-γ-release assay (QuantiFERON-TB Gold In-Tube), i.e., were classified as having latent tuberculosis infection. The median age was 26 years [interquartile range (IQR): 22–34 years] and 44/58 (76%) were female. All HIV-infected individuals were antiretroviral treatment (ART)-naive, with a median CD4 count of 591 cells/mm^3^ (IQR: 511–749).

All studies were conducted in a BSL-2+ laboratory environment under approval of the University of Cape Town’s Institutional Biosafety Committee (IBC007-2020). All samples were treated with 1% Triton-X100 (Sigma) for 60 min at room temperature to inactivate any potentially live virus in the samples (Remy et al., 2019).

### Enzyme-Linked Immunosorbent Assay

The ELISA protocol was adapted from a published protocol (Stadlbauer et al., 2020). Briefly, 96-well plates (Nunc MaxiSorp, Thermo Fisher) were coated at 4°C overnight with 50 μl of varying concentrations (1–4 μg/ml) of purified recombinant RBD or S1 proteins in PBS or bicarbonate buffer (both Sigma). The following day, plates were washed five times using an automated plate washer and incubated at room temperature in blocking solution [1% casein or 3% non-fat powder milk prepared in PBS with 0.1% Tween 20 (PBS-T)]. After 1 h, the blocking solution was discarded and 100 μl of serum, plasma, or saliva samples (at 1:50 dilution for sera/plasma and 1:10 for saliva) were added for 2 h at room temperature. Next, plates were washed five times and incubated with goat anti-human IgG (Fc-specific) peroxidase conjugate (1:5,000; IgG-HRP, Sigma), or goat anti-human IgA (α-chain specific), F(ab')_2_ fragment peroxidase conjugate (1:5,000; IgA-HRP, Sigma) or goat anti-human IgM peroxidase conjugate (1:2,000; IgM-HRP, Southern Biotech) for 1 h at room temperature. The plate was then developed using 100 μl O-phenylenediamine dihydrochloride (OPD; Sigma) for 12 min before the reaction was stopped with 50 μl 3 M hydrochloric acid (HCl, Sigma). The plates were read at 490 nm using a Versamax microplate reader (Molecular Devices) using SoftMax Pro software (version 5.3). A cutoff for positivity was set at 2 SD above the mean optical density (OD) of pre-pandemic samples. For determining endpoint titers, 2-fold serial dilutions were performed for 20 PCR+ samples and 40 pre-pandemic controls. Area under the curve (AUC) was determined and the positivity threshold was calculated as before, mean + 2 SD. All patient samples from SARS-CoV-2 RT-PCR+ volunteers were also analyzed using the anti-SARS-CoV-2 ELISA (IgG; Euroimmun), in an independent laboratory. Samples from 101 RT-PCR-volunteers were also tested on the same platform. The Euroimmun assay uses the S1 domain of the spike protein, expressed in mammalian cells. The assay was conducted according to the manufacturer’s instructions. Results were determined as a ratio of the OD signal of the samples to the average OD signal of calibrators, and are expressed as OD to calibrator ratio, as per the manufacturer’s recommendations. A ratio <0.8 was considered as negative, >0.8 to <1.1 as indeterminate or borderline, and >1.1 as positive.

### Statistical Analysis

Statistical analyses were performed in Prism (GraphPad, version 8). Nonparametric tests were used for all comparisons. The Friedman test with Dunn’s multiple comparison test was used for matched comparisons; the Mann-Whitney *U* unmatched and Wilcoxon matched pairs *t*-tests were used for unmatched and paired samples, respectively. Spearman Rank tests were used for all correlations. AUC was calculated in Prism. A value of *p* < 0.05 was considered statistically significant.

All data in this manuscript can be found in Supplementary Table 1.

## Results

### SARS-CoV-2 Antigen Expression in Plants

**Figure 1:**
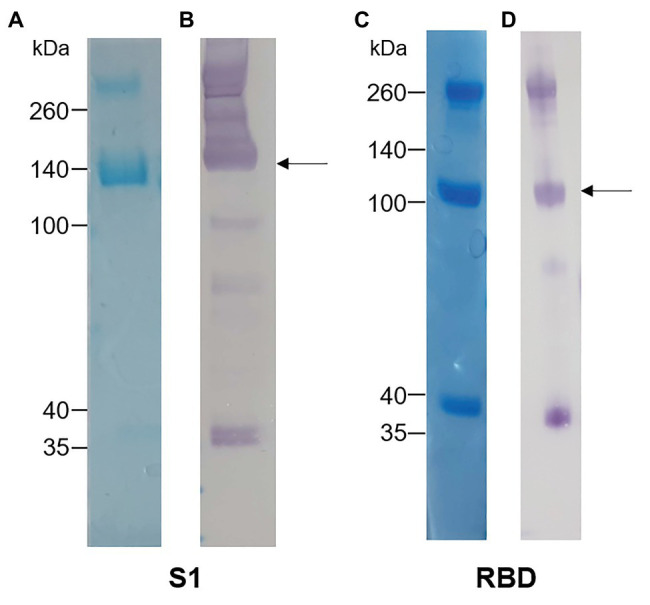
Analysis of plant-expressed severe acute respiratory syndrome coronavirus 2 (SARS-CoV-2) spike antigens after Protein A purification. **(A)** Coomassie-stained SDS-PAGE gel and **(B)** Western blot of S1-rabbit Fc fusion protein (2 μg of concentrated elution fraction). Lines on the left indicate molecular weight marker (Spectra Multicolor Broad range protein ladder) in kDa. The arrow indicates the expected size for recombinant S1 protein (~140 kDa). **(C)** Coomassie-stained SDS-PAGE gel and **(D)** Western blot of RBD-rabbit Fc fusion protein (5 μg of concentrated elution fraction). Arrows indicate expected size for RBD-rabbit Fc conjugate (~100 kDa).

The S1 and RBD portions of the Spike protein of SARS-CoV-2 were expressed in *N. benthamiana* as fusions to the rabbit IgG Fc tag. Western blot and SDS-PAGE analysis revealed expression of purified S1 (Figures 1A,B) and RBD (Figures 1C,D) at the expected protein sizes of ~140 and ~100 kDa, respectively. Higher molecular weight bands of ~280 and ~200 kDa indicated possible dimer formation of S1 and RBD, respectively. In addition, lower molecular weight bands indicated potentially multiple cleavage products of S1 and RBD in the preparations.

### Participant Description

Serum samples were collected from 77 volunteers who had previously tested positive for SARS-CoV-2 by PCR. The demographic and clinical characteristics of the participants are summarized in Table 1. Just over half the participants were female, and the median age was 39 years. The date of onset of symptoms was not available, but samples were taken a median of 6 weeks after SARS-CoV-2 PCR positivity. The majority of patients (79%) experienced mild or moderate COVID-19 disease. We also included 58 archived plasma samples from HIV-infected and uninfected individuals collected prior to the pandemic (2011–2012) as negative controls for our assay. For the commercial Euroimmun test kit, an additional 101 SARS-CoV-2 PCR negative participants were included as controls.

**Table 1:** Characteristics of Coronavirus disease 2019 (COVID-19) patients (*n* = 77).

Sex female, *n* (%)	42 (55)
Age (years)^a^	39 (29–50)
Time since positive PCR test (days)^a^	42 (29–52)
Disease severity, **n** (%)^b^	
Asymptomatic	5 (7)
Mild	23 (30)
Moderate	38 (49)
Severe	2 (3)

a Median and interquartile range.

b Not available for *n* = 9 participants.

### Optimization of the ELISA Assay

**Figure 2:**
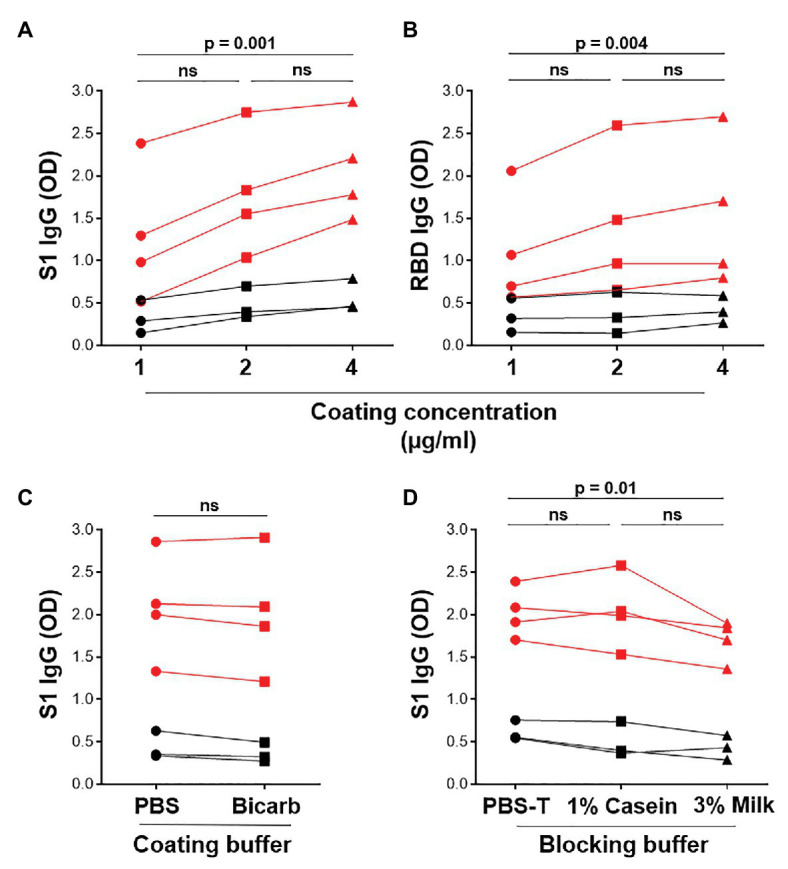
Optimization of ELISA antigen coating concentration, coating buffer, and blocking buffer. The effect of antigen coating concentration (1, 2, and 4 μg/ml) was tested for **(A)** S1 and **(B)** RBD, using serum samples from SARS-CoV-2 positive convalescent participants (*n* = 7). Statistical analyses were performed using the Friedman test with Dunn’s test for multiple comparisons. **(C)** Comparison of phosphate buffered saline (PBS) and bicarbonate buffer for coating viral antigens. Statistical analyses were performed using a Wilcoxon matched pair’s test. **(D)** The effect of different blocking solutions. Statistical analysis was performed using the Friedman test with Dunn’s test for multiple comparisons.

The in-house ELISA diagnostic assay in this study was developed from the published protocol (Stadlbauer et al., 2020). To establish a robust and sensitive in-house ELISA, we optimized several parameters, including S1 and RBD antigen coating concentration, as well as the coating and blocking buffers. Coating concentrations of 1, 2, and 4μg/ml S1 and RBD were compared for SARS-CoV-2-specific IgG detection in four SARS-CoV-2 convalescent volunteers and three pre-pandemic samples. Two and 4 μg/ml demonstrated a significantly higher reactivity than 1 μg/ml for both S1 and RBD (Figures 2A,B; *p* = 0.0005 and *p* = 0.004, respectively, using the Friedman test with Dunn’s test for multiple comparisons), with little increase in the background (negative control) signal. Thus, 2 μg/ml was selected for subsequent assays. Coating of ELISA plates with antigen in different coating buffers, namely PBS and bicarbonate buffer, was also assessed (Figure 2C). No differences were detected, so PBS was selected for our procedure. A comparison of the blocking buffers PBS with 0.1% Tween-20 (PBS-T), PBS-T with 1% casein and PBS-T with 3% non-fat milk powder was performed (Figure 2D). PBS-T with 1% casein was selected based on background signal and positivity trends. We also determined the optimal titer of secondary antibody IgG-HRP (1:5,000), as well as optimal serum dilution (1:50; data no shown).

### Plant-Produced S1 and RBD Proteins Are Suitable for ELISA Detection of SARS-CoV-2 Antibodies

In order to test whether plant-produced SARS-CoV-2 antigens were able to detect virus-specific antibodies from infected patients, we screened convalescent sera from 77 volunteers who had recovered from COVID-19. Individuals were tested for reactivity against both S1 and RBD antigens by a standard indirect ELISA based on a published protocol (Stadlbauer et al., 2020). Archived pre-pandemic plasma samples from 58 individuals, including 27 HIV-infected persons, were used to test the background reactivity to SARS-CoV-2 S1 and RBD. The threshold for positivity was set at 2 SD above the mean optical density (OD) of the pre-pandemic samples.

**Figure 3:**
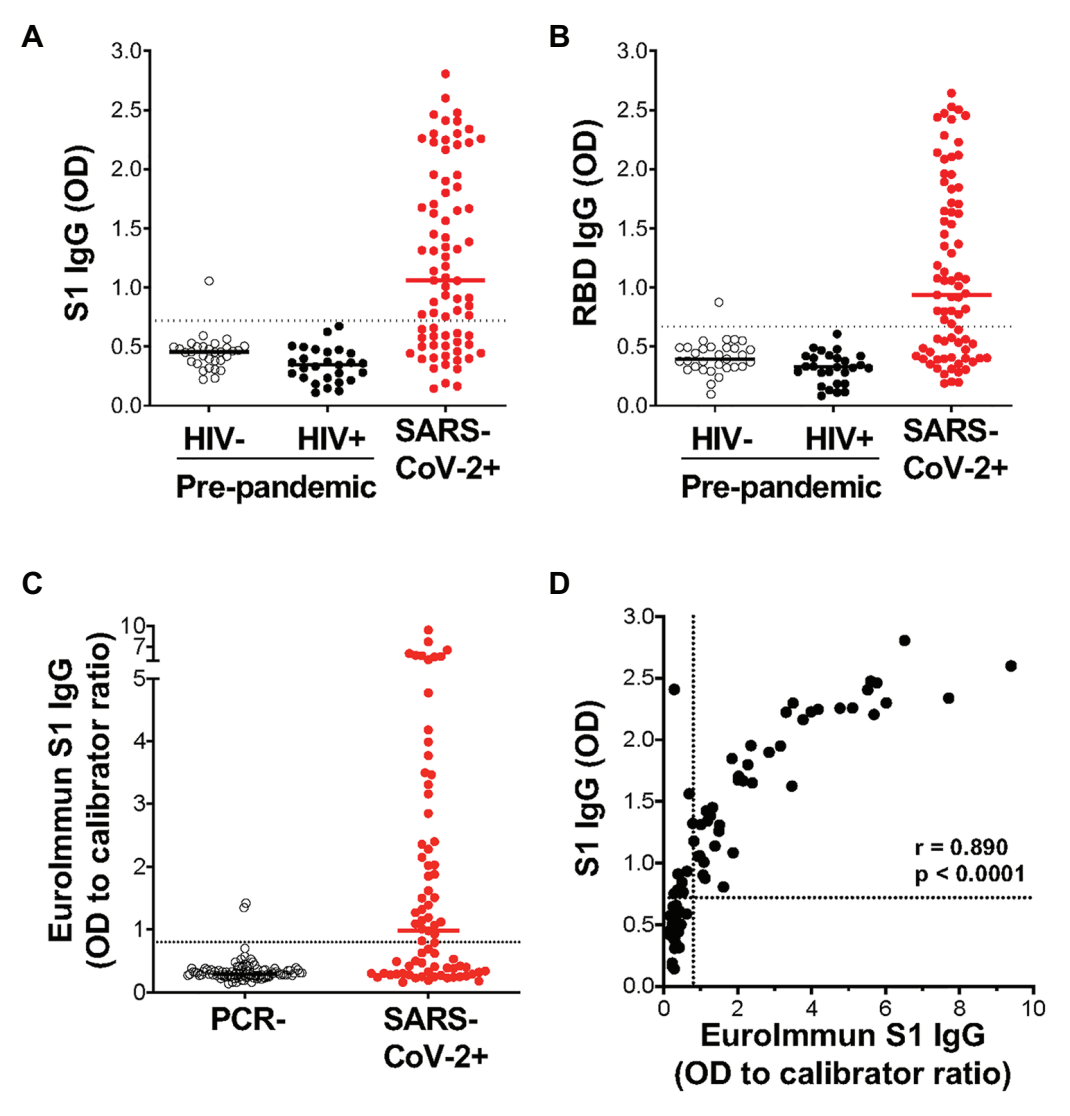
Detection of IgG using plant-expressed SARS-CoV-2 spike antigens in COVID-19 convalescent volunteers and pre-pandemic controls using an in-house ELISA. Reactivity to plant-expressed S1 **(A)** and RBD **(B)** in pre-pandemic samples from HIV-uninfected individuals (*n* = 31), HIV-infected individuals (*n* = 27), and SARS-CoV-2 PCR positive volunteers (*n* = 77). Dotted lines indicate threshold for positivity, calculated as the mean optical density (OD) + 2SD of the pre-pandemic samples. **(C)** Reactivity in Euroimmun IgG S1 of the same SARS-CoV-2 PCR positive volunteers (*n* = 77) and a set of PCR negative sera (*n* = 101). Results are expressed as OD to calibrator ratio, as per the manufacturer’s recommendations. The dotted line is at 0.8, above which samples are indeterminate or borderline (>0.8 and <1.1) or positive (>1.1). **(D)** Correlation of the OD values for S1-specific IgG in our in-house ELISA and the commercial Euroimmun IgG S1 ELISA assay. Statistical analyses were performed using a non-parametric Spearman rank correlation. Each dot represents one individual.

Of the 77 COVID-19 convalescent serum samples, 51 (66%) tested positive for SARS-CoV-2-specific IgG against S1, and 48 (62%) tested positive against RBD (Figures 3A,B). In contrast, only 1/58 pre-pandemic plasma samples showed reactivity above the positivity cutoff. As expected, S1 and RBD IgG OD values correlated strongly (*r* = 0.977; *p* < 0.0001; data not shown). In order to independently validate our results, the same PCR+ sera were run in a separate laboratory in a blinded manner, using a commercial IgG ELISA (Euroimmun) based on S1 antigen (Figure 3C). That assay included 101 PCR-sera, two of which were positive, and may represent false negative PCR tests. All samples that were positive by the commercial ELISA test for SARS-CoV-2 S1 antibodies were positive in our assay (42/77). We detected nine additional samples that were positive in our assay, two of which had high OD values well above our threshold for positivity, and six that were also positive for RBD-specific IgG. We demonstrated a strong and significant direct correlation for sample OD values between the two assays (*r* = 0.89, *p* < 0.0001, Spearman Rank test, Figure 3D). Of note, we found no association between SARS-CoV-2-specific IgG OD values and disease severity or days post PCR positivity (data not shown). Thus, our ELISA using plant-produced recombinant viral proteins performed similarly to a highly sensitive and specific commercial SARS-CoV-2 ELISA using S1 antigen from a mammalian expression system.

### Determination of Immunoglobulin Titers and Isotypes

**Figure 4:**
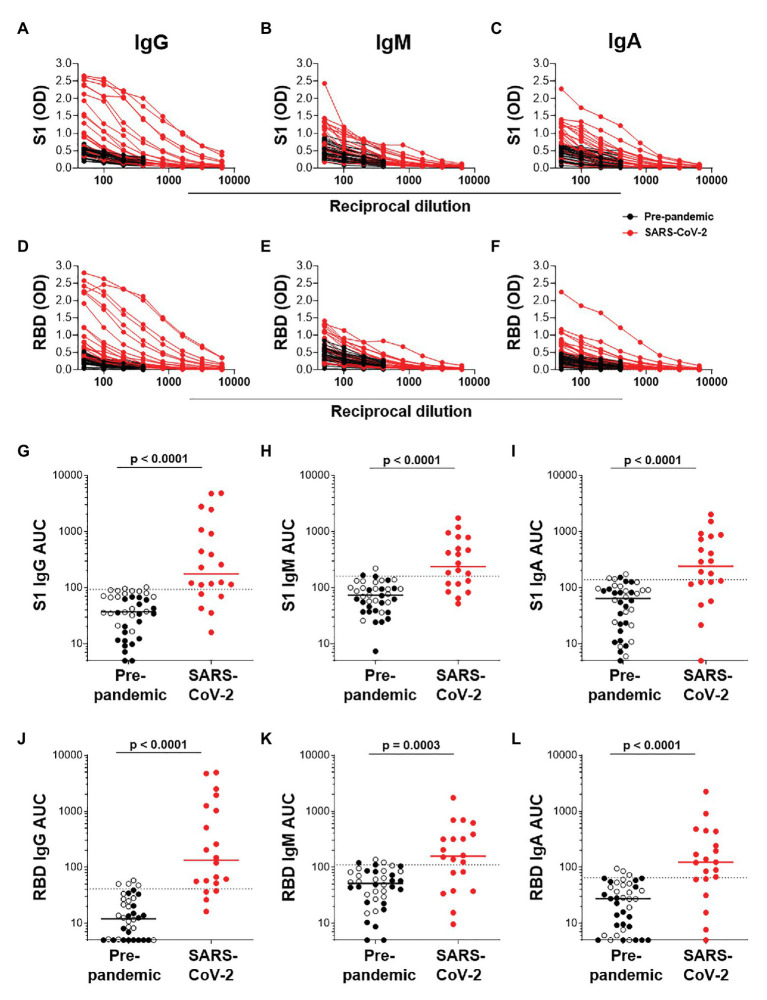
Semi-quantitative detection of S1- and RBD-specific IgG, IgM, and IgA. Two-fold dilution series of sera for detection of S1-specific IgG **(A)**, IgM **(B)**, and IgA **(C)** and RBD-specific IgG **(D)**, IgM **(E)**, and IgA **(F)**. COVID-19 convalescent volunteers (*n* = 20) are indicated in red, and pre-pandemic controls (*n* = 40) are indicated in black. **(G–I)** and **(J–L)**; Data from the same experiment as in **(A–C)** and **(D–F)**, respectively, but plotted as area under the curve (AUC). Horizontal lines represent median values. Dotted lines indicate the threshold for positivity. Statistical analyses were performed using a Mann-Whitney *U* test. A value of *p* < 0.05 was considered statistically significant.

We next determined the titers of SARS-CoV-2-specific IgG, IgM, and IgA responses in a subset of 20 SARS-CoV-2 convalescent serum samples and 40 pre-pandemic samples. Assays were performed on serially diluted samples (Figures 4A–F) to determine endpoint titers and AUC values for quantitative interrogation of the data (Figures 4G–L). S1-specific IgG was detected in sera of 15/20 individuals (75%), IgM in 13/20 (65%), and IgA in 12/20 (70%) of individuals (Figures 4G–I). The median AUCs of IgG, IgM, and IgA were significantly higher in convalescent individuals compared to pre-pandemic (*p* < 0.0001 for all, Mann-Whitney *U* test). Results for RBD-specific IgG were similar (Figures 4J–L). Interestingly, of the five SARS-CoV-2 convalescent sera that tested S1 IgG negative, three had S1-specific IgM and one had S1-specific IgA. Similarly, of the four samples negative for RBD-specific IgG, three were positive for IgM and one was double positive for IgM and IgA. Therefore, SARS-CoV-2 S1-specific antibodies were detected in 19/20 convalescent samples and RBD-specific antibodies in 20/20 samples.

**Figure 5:**
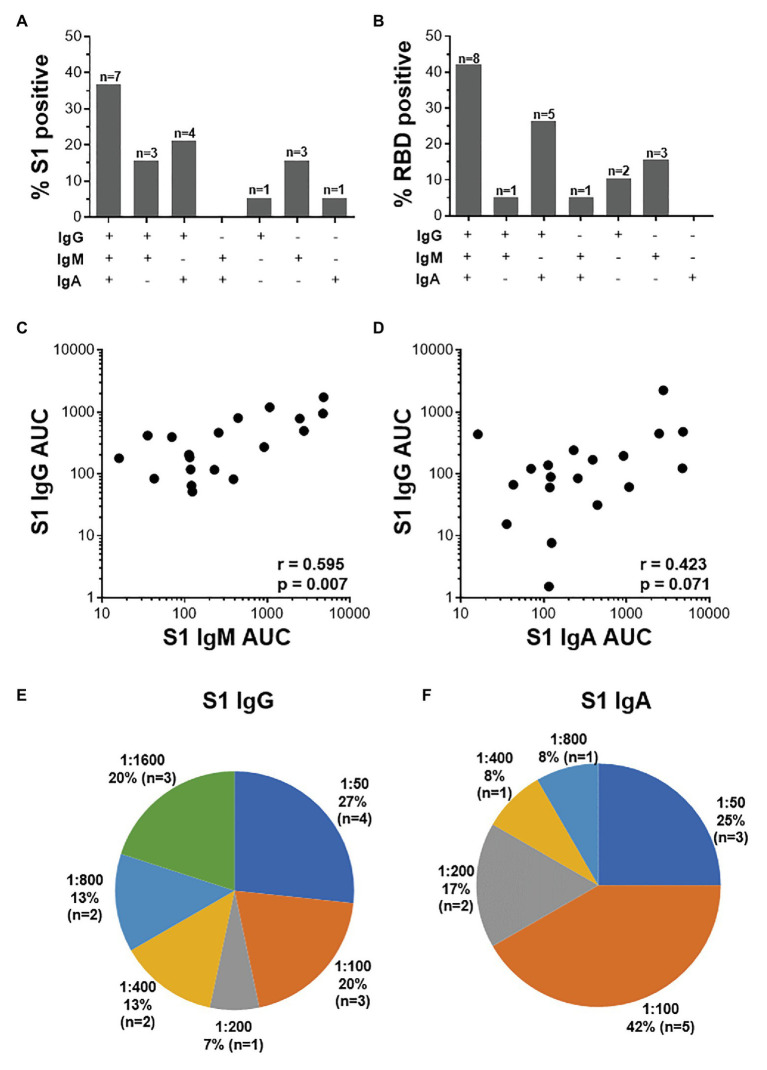
The relationship between IgG, IgM, and IgA responses to S1 and RBD SARS-CoV-2 antigens. **(A)** Proportions of COVID-19 convalescent volunteers mounting different combinations of IgG, IgM, and IgA specific for S1 (**A**; *n* = 19) and RBD (**B**; *n* = 20). Relationship between S1-specific IgG and IgM **(C)** and IgG and IgA **(D)**. Statistical analyses were performed using a non-parametric Spearman rank correlation. Proportion of convalescent volunteers with endpoint titers for IgG **(E)** and IgA **(F)** of 1:50, 1:100, 1:200, 1:400, 1:800, and 1:1,600.

Further examination of S1-specific antibody isotypes revealed that approximately one-third of individuals were positive for IgG, IgM, and IgA (*n* = 7/19), a smaller proportion had both IgG and IgM or IgG and IgA (*n* = 3 and 4, respectively), while some individuals were positive for only IgG (*n* = 1), IgM (*n* = 3), or IgA (*n* = 1; Figure 5A). RBD-specific isotypes gave similar results (Figure 5B). There was a significant correlation between S1-specific IgG and IgM (*r* = 0.595, *p* < 0.007, Spearman Rank test, Figure 5C) and anti-RBD (*r* = 0.045, *p* < 0.045; data not shown). S1-specific IgG showed a trend toward a correlation with IgA (*r* = 0.423, *p* = 0.07; Figure 5D), while RBD-specific IgG correlated significantly with IgA (*r* = 0.635, *p* < 0.003; data not shown). There was no correlation between IgM and IgA responses for either S1 or RBD (data not shown).

Endpoint titers for S1- and RBD-specific IgG, IgM, and IgA were determined. S1-specific IgG endpoint titers in 33% of the samples were high (20% at 1:1,600 and 13% at 1:800), 13% were moderate (1:400), and the majority (54%) of samples had low titers (27% at 1:50, 20% at 1:100, and 7% at 1:200; Figure 5E). S1-specific IgA titers were lower than IgG and only two individuals have a titer of 1:800 or 1:400 each, and the remaining 84% had low titers (=<1:200; Figure 5F). IgM titers for both S1 and RBD were all low (=<1:100; data not shown). RBD-specific titers for IgG and IgM were similar to those S1, with the exception of two donors who had titers of 1:3,200 (data not shown).

### Detection of SARS-CoV-2-Specific Antibodies in Saliva

**Figure 6:**
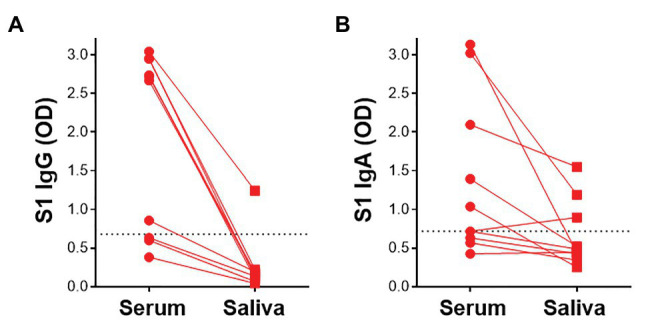
Detection of S1-specific antibodies in saliva. Comparison of paired serum and saliva S1-specific IgG **(A)** and IgA (**B**; *n* = 10). Dotted lines indicate the positivity threshold for serum.

Given that virus-specific serum antibodies were readily detectable using plant-produced SARS-CoV-2 antigens, we investigated the detection of salivary IgG and IgA using our assay. We compared antibody responses to SARS-CoV-2 antigens in paired saliva and serum from 10 participants. In these preliminary analyses, 1/7 samples that had detectable S1-specific serum IgG also demonstrated S1 IgG positivity in saliva (Figure 6A). Additionally, 2/5 IgA+ sera exhibited virus-specific IgA in saliva. An additional IgA+ sample was detected in saliva but absent from the serum (Figure 6B). This indicated that IgA was more readily detectable in saliva than IgG. Further analyses to determine robust thresholds for positivity of saliva immunoglobulins will be performed going forward. These preliminary results demonstrate the potential of our ELISA to detect antibodies to SARS-CoV-2 in saliva.

## Discussion

There is a critical need for the development of serological tests to detect SARS-CoV-2 antibodies. Population seroprevalence studies to estimate the extent of pandemic spread in communities, and studies defining protective immunity to SARS-CoV-2, all depend on reliable serological tests. In addition, serological assays are required for the development and evaluation of an effective vaccine. Ideally, such tests need to be cost-effective and easy to scale up to be beneficial in low-income settings. In this study, we describe the establishment of an indirect SARS-CoV-2 antibody ELISA using the S1 and RBD antigens of the spike protein of SARS-CoV-2 expressed in *N. benthamiana*. S protein domains were selected because they are highly immunogenic and the primary target for neutralizing antibodies (Berry et al., 2010; Chen et al., 2020). Using sera from convalescent volunteers with a PCR-confirmed past SARS-CoV-2 infection, we detected SARS-CoV-2-specific IgG, IgA, and IgM to viral S1 and RBD. Our results were highly concordant with a widely used, high sensitivity, and specificity commercial S1 IgG ELISA kit (Euroimmun).

A range of expression systems exist for the generation of the recombinant proteins required for serological assays. Plant protein expression systems have some advantages over more widely-used mammalian or insect cell systems, as they do not require expensive media or growth conditions (Shanmugaraj et al., 2020). They are also advantageous over bacterial or yeast systems in that they may support post-translational modifications similar to that of mammalian cell lines, and lack contaminating pathogens or endotoxins that pose a problem when purifying desired proteins (Maliga and Graham, 2004; Shanmugaraj et al., 2020). Lack of correct protein glycosylation and recombinant protein yield are cited as disadvantages to using plants to express protein. However, *N. benthamiana* is favored for protein expression due to its rapid generation of biomass, a defective post-transcriptional gene silencing system, and the extensive range of engineering strategies, including glycoengineering, that can be applied along its secretory pathway; all of which may overcome the challenge of low yield (Margolin et al., 2020). SARS-CoV-2 Spike is heavily glycosylated (22 sites), and different patterns of glycosylation between plant and mammalian expression systems may impact antigen recognition and thus test sensitivity (Rosales-Mendoza et al., 2020; Watanabe et al., 2020). The glycans in SARS-CoV-2 are made up of complex and high-mannose configurations; however, the structure of high-mannose glycans is conserved across eukaryotes (Capell et al., 2020), potentially mitigating differences between plant and mammalian-produced Spike. Furthermore, we used the S1 and RBD portions of Spike, which have fewer of the glycosylation sites present (7 and 1, respectively; Rosales-Mendosa et al., 2020) compared to full length Spike. Nonetheless, glycan-specific antibodies may be missed using plant-produced antigens and we may underestimate antibody reactivity in clinical samples. On the other hand, plant glycans, unlike those from mammalian cells, do not contain sialic acid (Bohlender et al., 2020). While the implications of this are not fully elucidated, sialic acid may shield epitopes (Galili, 2020), potentially resulting in increased reactivity to plant-produced proteins. Our data show comparable results to a commercial assay using mammalian-expressed Spike, suggesting that the effects of differential glycosylation and sialylation of antigens on assay sensitivity are not substantial; nevertheless, further studies are warranted. Thus, there is great potential to use plant-based expression systems for the rapid generation of serological assay reagents and even vaccines for pandemics, including the current global SARS-CoV-2 pandemic.

Using our ELISA with plant-derived recombinant viral proteins, we detected S1-specific IgG in 66.2% and RBD-specific IgG in 62.3% of individuals who had tested positive for SARS-CoV-2 by PCR in the past. Responses between the two protein fragments were highly correlated, as predicted, and the small difference in reactivity was not unexpected, given the greater number of epitopes in the larger S1 domain. Our sensitivity appears lower than that reported in the literature, with a seroprevalence of 90.1–100% in individuals confirmed to have been SARS-CoV-2-infected by PCR (Amanat et al., 2020; Beavis et al., 2020; Liu et al., 2020; Long et al., 2020), and a lower seroprevalence (65.8%) in those who were diagnosed <14 days before serological testing (Pollán et al., 2020). However, we obtained highly concordant results between our assay and a validated commercial ELISA. In fact, the reported manufacturer’s sensitivity of the Euroimmun S1-specific IgG ELISA is 94.4%. This suggests that the lack of S1-specific IgG detection from some recovered COVID-19 patients in our cohort is more likely due to low or absent S1-specific IgG antibody at the time of sampling, rather than a lack of sensitivity in our assay. Antibodies in SARS-CoV-2 infection predominantly target S and N, and a range of commercial and in-house immunoassays have been developed (Houlihan and Beale, 2020). While the sensitivity to detect infection using these antigens does not appear to differ during acute infection, reports are now emerging that S antibodies persist for longer than N antibodies (Grandjean et al., 2020; Fenwick et al., 2021), consistent with the original SARS infection (Chia et al., 2020). In addition to the waning of N antibodies in the post-acute/convalescent phase, Fenwick et al. (2021) also reported that trimeric S protein detected 9–31% more seropositives than monomeric S1 and N, indicating a considerable underestimation of true seroprevalence. That study included the Euroimmun S1 IgG ELISA used in the present study. We may thus have detected a higher seroprevalence in our sample set had we used a trimeric S protein as antigen, rather than monomeric S.

With regard to specificity, we detected IgG cross-reactivity to SARS-CoV-2 in 1/58 (1.7%) of pre-pandemic plasma samples from a cohort of HIV-infected and uninfected volunteers with latent TB infection, giving a specificity of 98.3%. Cross-reactive antibody responses, while lower in magnitude, have been reported in SARS-CoV-2 unexposed individuals (Khan et al., 2020), and likely result from past infections with common circulating hCoVs. Thus, our assay for SARS-CoV-2-specific IgG performs as well as a widely used commercial kit in terms of sensitivity and specificity, and is suitable for serological studies of humoral responses in the current pandemic.

Several factors may affect antibody detection after SARS-CoV-2 exposure. Timing of sampling is important, with IgM typically arising first, peaking 2–3 weeks after symptom onset (Long et al., 2020). IgG is typically detected after IgM in serum, peaking at roughly the same time (Huang et al., 2020). However, in SARS-CoV-2 infection, antibodies may not follow this typical pattern of seroconversion (Long et al., 2020; Seow et al., 2020) and seroconversion to a single Ig subclass has been described (Seow et al., 2020). SARS-CoV-2-specific antibodies are rapidly and readily induced after infection, but the kinetics may be influenced by multiple factors, such as cross-reactive serum antibodies as well as memory B cells from hCoVs (Hartley et al., 2020; Morgenlander et al., 2021). This appears similar to other hCoVs, including SARS-CoV and MERS-CoV (Huang et al., 2020). Interestingly, when investigating isotype responses in addition to IgG, we showed that a further 4/20 (20%) donors had S1-specific IgA or IgM. Thus, in our initial screen where 34% of individuals who had previously tested positive for SARS-CoV-2 by PCR had no detectable IgG responses, 20% may have had isotype responses other than IgG. A recent study showed that combined detection of IgG, IgM, and IgA increased the overall detection of SARS-CoV-2 antibodies, enabling better identification of infected individuals with low antibody levels (Faustini et al., 2020).

A further factor in detection of antibodies to SARS-CoV-2 is waning of the response over time, which has potentially important consequences for the duration of protective immunity and the risk of reinfection. One study showed a decrease in IgG in half of patients tested, calculating an overall half-life of 36 days for IgG (Ibarrondo et al., 2020). Waning of binding antibody responses to S and RBD has been reported soon after their peak, particularly IgM and IgA antibodies, but IgG responses have shown persistence for greater than 90 days post-illness onset (Seow et al., 2020; Wajnberg et al., 2020). A limitation of our study was that we did not have information on the date of COVID-19 symptom onset in our cohort, limiting our analyses to time post PCR positivity, which did not yield a relationship with antibody positivity or OD value. Additional factors that may also influence antibody generation and kinetics include disease severity, age, and comorbidities. We found no relationship between increasing disease severity and antibody positivity or OD value, likely due to the fact that the majority of our study participants had mild to moderate COVID-19.

We determined endpoint titers of binding antibodies to S1 and RBD in a subset of 20 convalescent participants in our cohort. Several studies have demonstrated that binding antibody titers against S correlate with neutralization capacity (Amanat et al., 2020; Okba et al., 2020; Premkumar et al., 2020). A recent study reporting S-specific IgG titers in almost 20,000 patients screened for eligibility as convalescent plasma donors demonstrated that 70% of IgG+ donors had high titers (>1:960) of antibodies (Wajnberg et al., 2020). Importantly, 100% of those with titers >2,880 exhibited neutralizing activity (ID_50_ of >1:10). Although we performed our study on a much smaller sample size, we detected titers of S1 or RDB-specific IgG of up to 1:3,200. However, the majority of donors (54%) had titers below 1:200, and only a third of samples had high titers >1:800. Unsurprisingly, IgA and IgM titers were lower than IgG titers, and did not exceed 1:800 for IgA and 1:400 for IgM. Further studies characterizing antibody titers in recovered COVID-19 patients in our setting are warranted.

Saliva is a non-invasive specimen that can be self-collected and thus represents an attractive sample type for large-scale sampling such as in seroprevalence studies. We demonstrate that our ELISA can detect SARS-CoV-2-specific IgG and IgA not only in serum, but also in saliva. Further optimization and validation will be required to establish the conditions for optimal detection of antibodies in saliva, including the use of pre-pandemic saliva samples. Recent studies have reported the detection of S-specific antibodies in saliva (Faustini et al., 2020; Randad et al., 2020). Faustini et al. (2020) suggested that the use of both serum and saliva samples increased the detection of SARS-CoV-2 antibody responses, reporting substantial discordance between the two sample types. Although preliminary, our results provide the basis for investigating the detection of SARS-CoV-2 antibodies in saliva using antigens expressed in plants.

In conclusion, our study demonstrates that recombinant SARS-CoV-2 proteins produced in plants enable the robust detection of SARS-CoV-2-specific antibodies. One of our aims was to develop a cost-effective serological assay for both large-scale seroepidemiology as well as research studies of SARS-CoV-2 humoral immunity. We achieved this by making use of plants for the production of viral antigens, which has the benefit of rapid scale-up, and sourcing reagents that were available locally and thus available at a lower cost. Our ELISA can be used to evaluate SARS-CoV-2 seroprevalence and describe the kinetics of the humoral immune response in infected individuals. Serological studies in a setting like ours, in South Africa, where comorbidities such as HIV and TB are highly prevalent, are underexplored and can benefit from this assay.

## Data Availability Statement

The raw data supporting the conclusions of this article will be made available by the authors, without undue reservation.

## Ethics Statement

The studies involving human participants were reviewed and approved by Faculty of Health Sciences Human Research Ethics Committee, University of Cape Town. The patients/participants provided their written informed consent to participate in this study.

## Author Contributions

WB, MM, MT, JW, FP, SB, EM, LS, and JB: conceived and designed the study and experiments. WS and IS: provided support and critical protocol review. MM, MT, JW, AD, SJ, NM, MG, FP, and SB: performed the experiments. MM, AD, SJ, NM, MG, SI, RN, RK, and WB: analyzed the data. FP, SI, RN, RK, MT, JW, MM, and WB: wrote the paper. All authors contributed to the article and approved the submitted version.

### Conflict of Interest

FP and SB are employed by Cape Bio Pharms.

The remaining authors declare that the research was conducted in the absence of any commercial or financial relationships that could be construed as a potential conflict of interest.

## References

[ref1] Amanat F. Stadlbauer D. Strohmeier S. Nguyen T. H. O. Chromikova V. McMahon M.. (2020). A serological assay to detect SARS-CoV-2 seroconversion in humans. Nat. Med. 26, 1033–1036. 10.1038/s41591-020-0913-5, PMID: 32398876 PMC8183627

[ref3] Beavis K. G. Matushek S. M. Abeleda A. P. F. Bethel C. Hunt C. Gillen S.. (2020). Evaluation of the EUROIMMUN anti-SARS-CoV-2 ELISA assay for detection of IgA and IgG antibodies. J. Clin. Virol. 129:104468. 10.1016/j.jcv.2020.104468, PMID: 32485620 PMC7255182

[ref4] Berry J. D. Hay K. Rini J. M. Yu M. Wang L. Plummer F. A.. (2010). Neutralizing epitopes of the SARS-CoV S-protein cluster independent of repertoire, antigen structure or mAb technology. MAbs 2, 53–66. 10.4161/mabs.2.1.10788, PMID: 20168090 PMC2828578

[ref5] Bohlender L. L. Parsons J. Hoernstein S. N. W. Rempfer C. Ruiz-Molina N. Lorenz T.. (2020). Stable protein sialylation in physcomitrella. Front. Plant Sci. 11:610032. 10.3389/fpls.2020.610032, PMID: 33391325 PMC7775405

[ref6] Capell T. Twyman R. M. Armario-Najera V. Ma J. K. Schillberg S. Christou P. (2020). Potential applications of plant biotechnology against SARS-CoV-2. Trends Plant Sci. 25, 635–643. 10.1016/j.tplants.2020.04.009, PMID: 32371057 PMC7181989

[ref7] Chan J. F. W. Yuan S. Kok K. H. To K. K.-W. Chu H. Yang J.. (2020). A familial cluster of pneumonia associated with the 2019 novel coronavirus indicating person-to-person transmission: a study of a family cluster. Lancet 395, 514–523. 10.1016/S0140-6736(20)30154-9, PMID: 31986261 PMC7159286

[ref8] Chen W. H. Hotez P. J. Bottazzi M. E. (2020). Potential for developing a SARS-CoV receptor-binding domain (RBD) recombinant protein as a heterologous human vaccine against coronavirus infectious disease (COVID)-19. Hum. Vaccines Immunother. 16, 1239–1242. 10.1080/21645515.2020.1740560, PMID: 32298218 PMC7482854

[ref9] Chia W. N. Tan C. W. Foo R. Kang A. E. Z. Peng Y. Sivalingam V.. (2020). Serological differentiation between COVID-19 and SARS infections. Emerg. Microbes Infect. 9, 1497–1505. 10.1080/22221751.2020.1780951, PMID: 32529906 PMC7473126

[ref10] Faustini S. E. Jossi S. E. Perez-Toledo M. Shields A. Allen J. D. Watanabe Y.. (2020). Detection of antibodies to the SARS-CoV-2 spike glycoprotein in both serum and saliva enhances detection of infection. medRxiv [Preprint]. 10.1101/2020.06.16.20133025

[ref11] Fenwick C. Croxatto A. Coste A. T. Pojer F. André C. Pellaton C.. (2021). Changes in SARS-CoV-2 spike versus nucleoprotein antibody responses impact the estimates of infections in population-based seroprevalence studies. J. Virol. 95:e01828–20. 10.1128/JVI.01828-20, PMID: 33144321 PMC7925109

[ref12] Galili U. (2020). Amplifying immunogenicity of prospective Covid-19 vaccines by glycoengineering the coronavirus glycan-shield to present alpha-gal epitopes. Vaccine 38, 6487–6499. 10.1016/j.vaccine.2020.08.032, PMID: 32907757 PMC7437500

[ref13] Grandjean L. Saso A. Torres Ortiz A. Lam T. Hatcher J. Thistlethwayte R.. (2020). Long-term persistence of spike antibody and predictive modeling of antibody dynamics following infection with SARS-CoV-2. medRxiv [Preprint]. 10.1101/2020.11.20.20235697

[ref14] Hartley G. E. Edwards E. S. J. Aui P. M. Varese N. Stojanovic S. McMahon J.. (2020). Rapid generation of durable B cell memory to SARS-CoV-2 spike and nucleocapsid proteins in COVID-19 and convalescence. Sci. Immunol. 5:eabf8891. 10.1126/sciimmunol.abf8891, PMID: 33443036 PMC7877496

[ref15] Houlihan C. F. Beale R. (2020). The complexities of SARS-CoV-2 serology. Lancet Infect. Dis. 20, 1350–1351. 10.1016/S1473-3099(20)30699-X, PMID: 32979317 PMC7511169

[ref16] Huang A. T. Garcia-Carreras B. Hitchings M. D. T. Yang B. Katzelnick L. C. Rattigan S. M.. (2020). A systematic review of antibody mediated immunity to coronaviruses: kinetics, correlates of protection, and association with severity. Nat. Commun. 11:4704. 10.1038/s41467-020-18450-4, PMID: 32943637 PMC7499300

[ref17] Ibarrondo F. J. Fulcher J. A. Goodman-Meza D. Elliott J. Hofmann C. Hausner M. A.. (2020). Rapid decay of anti-SARS-CoV-2 antibodies in persons with mild Covid-19. N. Engl. J. Med. 383, 1085–1087. 10.1056/NEJMc2025179, PMID: 32706954 PMC7397184

[ref18] Kapila J. De Rycke R. Van Montagu M. Angenon G. (1997). An Agrobacterium-mediated transient gene expression system for intact leaves. Plant Sci. 122, 101–108. 10.1016/S0168-9452(96)04541-4

[ref19] Khan S. Nakajima R. Jain A. Assis R. R. Jasinskas A. Obiero J. M.. (2020). Analysis of serologic cross-reactivity between common human coronaviruses and SARS-CoV-2 using coronavirus antigen microarray. bioRxiv [Preprint]. 10.1101/2020.03.24.006544

[ref20] Letko M. Marzi A. Munster V. (2020). Functional assessment of cell entry and receptor usage for SARS-CoV-2 and other lineage B betacoronaviruses. Nat. Microbiol. 5, 562–569. 10.1038/s41564-020-0688-y, PMID: 32094589 PMC7095430

[ref21] Li F. (2016). Structure, function, and evolution of coronavirus spike proteins. Annu. Rev. Virol. 3, 237–261. 10.1146/annurev-virology-110615-042301, PMID: 27578435 PMC5457962

[ref22] Liu W. Liu L. Kou G. Zheng Y. Ding Y. Ni W.. (2020). Evaluation of nucleocapsid and spike protein-based enzyme-linked immunosorbent assays for detecting antibodies against SARS-CoV-2. J. Clin. Microbiol. 58, e00461–20. 10.1128/JCM.00461-20, PMID: 32229605 PMC7269413

[ref23] Long Q.-X. Liu B.-Z. Deng H.-J. Wu G.-C. Deng K. Chen Y.. (2020). Antibody responses to SARS-CoV-2 in patients with COVID-19. Nat. Med. 26, 845–848. 10.1038/s41591-020-0897-1, PMID: 32350462

[ref24] Lu R. Zhao X. Li J. Niu P. Yang B. Wu H.. (2020). Genomic characterisation and epidemiology of 2019 novel coronavirus: implications for virus origins and receptor binding. Lancet 395, 565–574. 10.1016/S0140-6736(20)30251-8, PMID: 32007145 PMC7159086

[ref25] Maclean J. Koekemoer M. Olivier A. J. Stewart D. Hitzeroth I. I. Rademacher T.. (2007). Optimization of human papillomavirus type 16 (HPV-16) L1 expression in plants: comparison of the suitability of different HPV-16 L1 gene variants and different cell-compartment localization. J. Gen. Virol. 88, 1460–1469. 10.1099/vir.0.82718-0, PMID: 17412974

[ref26] Maliga P. Graham I. (2004). Plant biotechnology: molecular farming and metabolic engineering promise a new generation of high-tech crops. Curr. Opin. Plant Biol. 7, 149–151. 10.1016/j.pbi.2004.01.016, PMID: 15003214

[ref27] Margolin E. A. Strasser R. Chapman R. Williamson A.-L. Rybicki E. P. Meyers A. E. (2020). Engineering the plant secretory pathway for the production of next-generation pharmaceuticals. Trends Biotechnol. 38, 1034–1044. 10.1016/j.tibtech.2020.03.004, PMID: 32818443

[ref28] Morgenlander W. R. Henson S. N. Monaco D. R. Chen A. Littlefield K. Bloch E. M.. (2021). Antibody responses to endemic coronaviruses modulate COVID-19 convalescent plasma functionality. J. Clin. Invest. 11:146927. 10.1172/JCI146927, PMID: 33571169 PMC8011893

[ref29] Neuman B. W. Kiss G. Kunding A. H. Bhella D. Baksh M. F. Connelly S.. (2011). A structural analysis of M protein in coronavirus assembly and morphology. J. Struct. Biol. 174, 11–22. 10.1016/j.jsb.2010.11.021, PMID: 21130884 PMC4486061

[ref30] Okba N. Muller M. Li W. Wang C. Geurts vanKessel C. Corman V.. (2020). SARS-CoV-2 specific antibody responses in COVID-19 patients. medRxiv [Preprint]. 10.1101/2020.03.18.20038059

[ref31] Pogrebnyak N. Golovkin M. Andrianov V. Spitsin S. Smirnov Y. Egolf R.. (2005). Severe acute respiratory syndrome (SARS) S protein production in plants: development of recombinant vaccine. Proc. Natl. Acad. Sci. U. S. A. 102, 9062–9067. 10.1073/pnas.0503760102, PMID: 15956182 PMC1157057

[ref32] Pollán M. Pérez-Gómez B. Pastor-Barriuso R. Oteo J. Hernán M. A. Pérez-Olmeda M.. (2020). Prevalence of SARS-CoV-2 in Spain (ENE-COVID): a nationwide, population-based seroepidemiological study. Lancet 396, 535–544. 10.1016/S0140-6736(20)31483-5, PMID: 32645347 PMC7336131

[ref33] Premkumar L. Segovia-Chumbez B. Jadi R. Martinez D. R. Raut R. Markmann A.. (2020). The receptor binding domain of the viral spike protein is an immunodominant and highly specific target of antibodies in SARS-CoV-2 patients. Sci. Immunol. 5:eabc8413. 10.1126/sciimmunol.abc8413, PMID: 32527802 PMC7292505

[ref34] Randad P. R. Pisanic N. Kruczynski K. Manabe Y. C. Thomas D. Pekosz A.. (2020). COVID-19 serology at population scale SARS-CoV-2-specific antibody responses in saliva. medRxiv [Preprint]. 10.1101/2020.05.24.20112300 PMC7771435 33067270

[ref35] Remy M. M. Alfter M. Chiem M. N. Barbani M. T. Engler O. B. Suter-Riniker F. (2019). Effective chemical virus inactivation of patient serum compatible with accurate serodiagnosis of infections. Clin. Microbiol. Infect. 25, 907.e7–907.e12. 10.1016/j.cmi.2018.10.016, PMID: 30391583 PMC7128130

[ref36] Rogers T. F. Zhao F. Huang D. Beutler N. Burns A. He W.. (2020). Isolation of potent SARS-CoV-2 neutralizing antibodies and protection from disease in a small animal model. Science 369, 956–963. 10.1126/science.abc7520, PMID: 32540903 PMC7299280

[ref500] Rosales-Mendoza S. Marquez-Escobar V. A. Gonzalez-Ortega O. Nieto-Gomez R. Arévalo-Villalobos J. I. (2020). What does plant-based vaccine technology offer to the fight against COVID-19? Vaccines (Basel) 8:183. 10.3390/vaccines8020183 PMC7349371 32295153

[ref37] Seow J. Graham C. Merrick B. Acors S. Steel K. J. A. Hemmings O.. (2020). Longitudinal evaluation and decline of antibody responses in SARS-CoV-2 infection. medRxiv [Preprint]. 10.1101/2020.07.09.20148429

[ref38] Shanmugaraj B. Malla A. Phoolcharoen W. (2020). Emergence of novel coronavirus 2019-nCoV: need for rapid vaccine and biologics development. Pathogens 9:148. 10.3390/pathogens9020148, PMID: 32098302 PMC7168632

[ref40] Stadlbauer D. Amanat F. Chromikova V. Jiang K. Strohmeier S. Arunkumar G. A.. (2020). SARS-CoV-2 seroconversion in humans: a detailed protocol for a serological assay, antigen production, and test setup. Curr. Protoc. Microbiol. 57:e100. 10.1002/cpmc.100, PMID: 32302069 PMC7235504

[ref41] Su S. Wong G. Shi W. Liu J. Lai A. C. K. Zhou J.. (2016). Epidemiology, genetic recombination, and pathogenesis of coronaviruses. Trends Microbiol. 24, 490–502. 10.1016/j.tim.2016.03.003, PMID: 27012512 PMC7125511

[ref42] Tan W. Zhao X. Ma X. Wang W. Niu P. Xu W.. (2020). A novel coronavirus genome identified in a cluster of pneumonia cases—Wuhan, China 2019−2020. China CDC Wkly. 2, 61–62. 10.46234/ccdcw2020.017 PMC8393069 34594763

[ref43] Tortorici M. A. Veesler D. (2019). Structural insights into coronavirus entry. Adv. Virus. Res. 105, 93–116. 10.1016/bs.aivir.2019.08.002 31522710 PMC7112261

[ref44] Wajnberg A. Amanat F. Firpo A. Altman D. R. Bailey M. J. Mansour M.. (2020). SARS-CoV-2 infection induces robust, neutralizing antibody responses that are stable for at least three months. medRxiv [Preprint]. 10.1101/2020.07.14.20151126

[ref45] Walls A. C. Park Y. J. Tortorici M. A. Wall A. McGuire A. T. Veesler D. (2020). Structure, function, and antigenicity of the SARS-CoV-2 spike glycoprotein. Cell 181, 281.e6–292.e6. 10.1016/j.cell.2020.02.058, PMID: 32155444 PMC7102599

[ref46] Watanabe Y. Allen J. D. Wrapp D. McLellan J. S. Crispin M. (2020). Site-specific glycan analysis of the SARS-CoV-2 spike. Science 369, 330–333. 10.1126/science.abb9983, PMID: 32366695 PMC7199903

[ref47] Wrapp D. Wang N. Corbett K. S. Goldsmith J. A. Hsieh C.-L. Abiona O.. (2020). Cryo-EM structure of the 2019-nCoV spike in the prefusion conformation. Science 367, 1260–1263. 10.1126/science.abb2507, PMID: 32075877 PMC7164637

[ref48] Yamamoto T. Hoshikawa K. Ezura K. Okazawa R. Fujita S. Takaoka M.. (2018). Improvement of the transient expression system for production of recombinant proteins in plants. Sci. Rep. 8, 1–10. 10.1038/s41598-018-23024-y, PMID: 29555968 PMC5859073

[ref49] Yin J. Li G. Ren X. Herrler G. (2007). Select what you need: a comparative evaluation of the advantages and limitations of frequently used expression systems for foreign genes. J. Biotechnol. 127, 335–347. 10.1016/j.jbiotec.2006.07.012, PMID: 16959350

[ref51] Zhu N. Zhang D. Wang W. Li X. Yang B. Song J.. (2020). A novel coronavirus from patients with pneumonia in China, 2019. N. Engl. J. Med. 382, 727–733. 10.1056/NEJMoa2001017, PMID: 31978945 PMC7092803

